# Prognostic Value of Concurrent Expression of C-MYC and BCL2 in Intravascular Large B-Cell Lymphoma: A 10-Year Retrospective Study

**DOI:** 10.1155/2020/1350820

**Published:** 2020-05-22

**Authors:** Paisarn Boonsakan, Wimolsiri Iamsumang, Pichika Chantrathammachart, Pamela Chayavichitsilp, Poonkiat Suchonwanit, Suthinee Rutnin

**Affiliations:** ^1^Department of Pathology, Faculty of Medicine Ramathibodi Hospital, Mahidol University, Bangkok, Thailand; ^2^Division of Dermatology, Department of Internal Medicine, Faculty of Medicine Ramathibodi Hospital, Mahidol University, Bangkok, Thailand; ^3^Division of Hematology, Department of Internal Medicine, Faculty of Medicine Ramathibodi Hospital, Mahidol University, Bangkok, Thailand

## Abstract

**Background:**

Intravascular large B-cell lymphoma (IVLBCL) is a variant of extranodal diffuse large B-cell lymphoma (DLBCL), characterized by the presence of a B-lymphoma cell in the lumina of small blood vessels or capillaries. Due to its extremely variable clinical manifestations, IVLBCL typically results in a delayed diagnosis and poor disease prognosis. Skin biopsy, particularly random skin biopsy, has shown a potential role in the diagnosis of IVLBCL. However, information of clinicopathological features in patients with IVLBCL diagnosed by skin biopsy is limited.

**Objectives:**

To study the clinicopathological features in relation to immunohistochemical features and to identify prognostic factors in IVLBCL patients diagnosed by skin biopsy.

**Materials and Methods:**

Clinical characteristics; laboratory, histological, and immunohistochemical findings; and therapeutic response of all biopsy-confirmed IVLBCL patients during the years 2008-2017 were retrospectively reviewed.

**Results:**

The mean age was 67.4 (±9.8) years. Fever was the most common presenting symptom, accounting for 64.7%. Cutaneous and bone marrow involvement was found in 23.5% and 35.3% of patients, respectively. Patients receiving R-CHOP showed more favorable therapeutic outcome. C-MYC/BCL2 double expressors showed significantly higher incidence rate to mortality compared with nondouble expressors (*p* = 0.042). One-year and two-year overall survival rates were 67.2% and 53.8%, respectively.

**Conclusions:**

Skin biopsy is an effective diagnostic method for IVLBCL. Concurrent expression of C-MYC and BCL2 may be a useful prognostic indicator and should be performed in order to predict the prognosis in IVLBCL patients.

## 1. Introduction

Intravascular large B-cell lymphoma (IVLBCL), first described as “angioendotheliomatosis proliferans systemisata” [1], is a rare variant of extranodal diffuse large B-cell lymphoma (DLBCL). It is characterized by the presence of lymphoma cells in the lumina of small blood vessels and an aggressive clinical course involving multiple organs [2, 3]. Two forms have been reported, including a “Classical form” or “Western form” with cutaneous and neurological manifestations, and “Asian form” with multiorgan failure, hepatosplenomegaly, pancytopenia, and hemophagocytic syndrome [4–6]. In one large study, fever was the most common presenting symptom followed by cutaneous and neurologic manifestations. However, abdominal pain, fatigue, weight loss, and dyspnea were also noted [5]. Given the fact that IVLBCL can present with a wide range of signs and symptoms depending on organ involvement, diagnosis and treatment are often delayed, resulting in poor disease prognosis [2]. Nonetheless, random skin biopsy (RSB) has been proposed as a useful diagnostic tool in IVLBCL by Gill et al. [7]. Subsequent studies also confirmed the potential of RSB in establishing the diagnosis of IVLBCL [8–14].

In DLBCL, not otherwise specified (NOS), immunohistochemistry (IHC) has been utilized as a tool for subgroup classification and prognostic prediction. Classifying by immunohistochemical expression, poor prognostic factors of DLBCL include nongerminal center B-cell (non-GCB) variant, C-MYC and BCL2 double expressor, positive C-MYC, positive BCL2, positive CD5, positive CD30, and positive P53 [15]. Likewise, determining factors linking to poor prognosis of IVLBCL is crucial, as it would allow physicians to be more aggressive in their management plans. Nevertheless, in IVLBCL, evidence in relationships between clinical features as well as immunohistochemistry and disease prognosis is limited. Therefore, the objective of this study was to determine clinicopathological features in relation to immunohistochemical features and to identify prognostic factors in intravascular large B-cell lymphoma (IVLBCL) patients diagnosed by skin biopsy.

## 2. Materials and Methods

### 2.1. Patient Selection and Data Collection

The medical records of all patients with IVLBCL diagnosed by skin biopsy at a university-based hospital (Ramathibodi Hospital, Mahidol University, Bangkok, Thailand) from January 2008 to December 2017 were retrospectively reviewed. Individuals with histologic diagnosis of IVLBCL from skin biopsy were included. Patients with incomplete data or unavailable histologic confirmation were excluded. In total, the medical records of 17 IVLBCL patients were retrieved and analyzed. The study protocol and medical record form were approved by the Mahidol University Institutional Review Board (Protocol number 08-60-08). Information regarding demographic data, clinical characteristics, laboratory investigation, treatment regimen, therapeutic outcome, and time from diagnosis to complete remission or death was collected.

### 2.2. Histopathology and Immunohistochemistry

In order to obtain the adequate depth and size of tissue, incisional skin biopsy of approximately 1 cm length, 0.5 cm width, and deep enough to include subcutaneous fat was performed. Three incisional skin biopsies were reviewed in all patients. If patients had no skin lesions, random skin biopsy was performed on 3 separate fat-bearing areas including both thighs and lower abdomen. However, if skin lesions were present, lesional skin biopsy with the same method was conducted on that area. Available formalin-fixed paraffin-embedded tissue blocks from 17 patients underwent standard pathologic procedure and were then reviewed by one hematopathologist and one dermatopathologist.

The avidin-biotin complex immunoperoxidase technique was utilized for immunohistochemical studies. CD3 (Dako Clone Polyclonal Rabbit, Glostrup, Denmark) and CD20 (Dako Clone L26, Glostrup, Denmark) were used as stains in all patients for diagnostic purposes. Antibodies including CD5 (Leica Clone 4C7), CD10 (Dako Clone 56C6), CD30 (Dako Clone Ber-H2), BCL2 (Dako Clone 124), BCL6 (Leica Clone 564), MUM1 (Dako Clone MUM1p), C-MYC (Ventana Clone Y69), and P53 (Dako Clone DO-7) were used in 15 patients to determine immunohistochemical findings linked to prognosis. Sections from 2 patients were not performed due to unavailable formalin-fixed paraffin-embedded tissue blocks (patient number 2) and sparse lymphoma cells in the sections (patient number 6). The cut-off values for a positive result were ≥30% positive cells for all markers except for C-MYC (≥40%) and BCL2 (≥50%) [3, 16, 17].

### 2.3. Statistical Analyses

Statistical analyses were conducted by STATA statistical software version 14 (StataCorp LP, College Station, TX, USA). To compare data between the two groups, Pearson's Chi-squared test or Fisher's exact test were applied for categorical variables while Student's *t*-test and Wilcoxon rank-sum test were utilized for continuous variables with normal and nonnormal distribution, respectively. An estimate of the actual time-at-risk to complete remission and mortality was performed using person-time analysis. Kaplan-Meier method was used to determine median survival time and overall survival proportion. Test for difference in survival function between the 2 groups with different histochemical expression was done by log-rank test and multiple Cox regression. Statistical significance was considered when *p* value < 0.05.

## 3. Results

### 3.1. Demography and Clinical Characteristics

Clinical characteristics are summarized in Table 1. The mean age of onset was 67.4 (±9.8) with slight male predominance (male : female ratio =1.4 : 1). In 17 patients, fever was reported as the most common presenting symptom, accounting for 64.7% (11 patients), followed by neurological symptoms 23.5% (4 patients), abdominal pain and jaundice 5.9% (1 patient), and anorexia 5.9% (1 patient). The duration of fever ranged from 1 to 12 weeks with a median of 8 weeks. Among 4 patients with neurological manifestations, 2 patients presented with alteration of consciousness, 1 patient with dementia, and one with progressive paralysis. B symptoms including fever, weight loss, and night sweat were found in 22.2% (2/9 patients).

**Figure 1:**
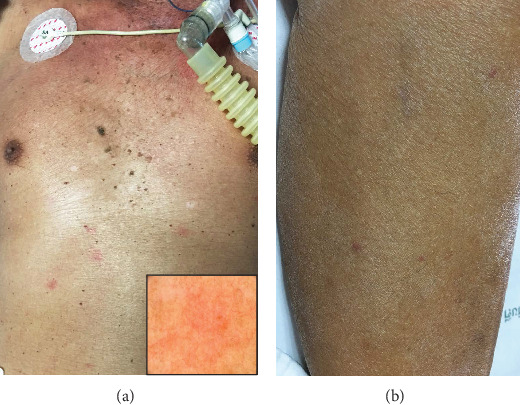
(a) Multiple discrete blanchable erythematous macules and patches with telangiectasia (inset) on the chest and abdomen. (b) Few discrete partially blanchable erythematous papules on the leg.

**Figure 2:**
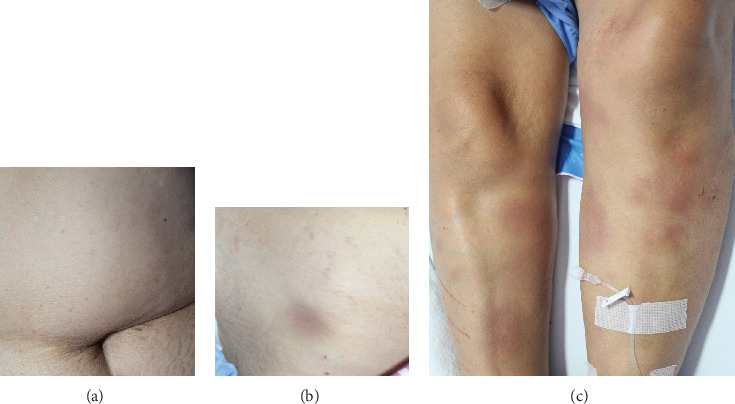
Multiple purpuric macules and patches on the abdomen (a, b) and both lower legs (c).

For organ-specific symptoms, neurological abnormalities were the most common, accounting for 41.2% or 7 out of 17 patients (3 patients developed neurological symptoms during the hospital stay). Hepatomegaly or splenomegaly was seen in 29.4% (5/17 patients), whereas dyspnea and cutaneous manifestations were found in 23.5% (4/17 patients). The morphology of skin lesions was variable including ecchymoses (patient number 7), blanchable erythematous macules and patches with telangiectasia (patient number 11, Figure 1(a)), and erythematous papules (patient number 15, Figure 1(b)). Patient number 12 initially had no cutaneous lesions and underwent RSB but, later on, developed multiple purpuric macules and patches on the abdomen and both lower legs (Figures 2(a)–2(c)) that were histologically proven to be IVLBCL. Lower extremities appeared to be the most common site (4 patients) while 2 patients had lesions on the trunk. No significant lymphadenopathy was noted.

### 3.2. Laboratory Investigation and Other Diagnostic Testing

Almost all patients (15 out of 17 patients, 88.2%) had mild anemia, while leukopenia and leukocytosis were found in 29.4% (5/17) and 17.7% (3/17), respectively. Thrombocytopenia was noted in 52.9% (9/17 of patients). Liver enzymes including AST and ALT were elevated in 75.0% and 43.8%, respectively. Increased sera LDH and ferritin were seen in all patients with a median level of 848 U/L (317-6,763 U/L) and 4629 ng/mL (343-35,766 ng/mL), respectively. Table 1 shows the international prognostic index or IPI score at time of diagnosis with the mean of 4.27 (±0.59).

In terms of other diagnostic testing, one patient (patient number 2) showed an abnormal chest radiography and a bronchoalveolar lavage showed atypical large B-lymphoid cells with CD20 positivity. One patient (patient number 11) suffered from pansinusitis and the diagnosis of IVLBCL was histologically confirmed. In patient number 13 who presented with dementia, a brain MRI revealed multiple enhancing lesions scattered throughout the cerebral hemisphere, mid-pons, and cerebellum. IVLBCL was diagnosed by the presence of atypical large cells in the vascular lumina from brain biopsy. In patient number 6 who presented with fever, an abdominal CT scan showed multiple wedge-shaped hypodense lesions suspected for splenic infarction from IVLBCL without definite tissue biopsy. Of all, 6 patients (35.3%) had bone marrow (BM) involvement which is defined by the presence of lymphoma cells in bone marrow biopsy. Although the criteria for hemophagocytic syndrome could not be evaluated due to missing data, 3 patients were found to have hemophagocytosis in the bone marrow biopsy.

### 3.3. Histopathology and Immunohistochemistry

**Figure 3:**
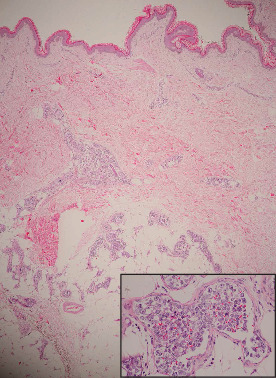
Infiltrates of large atypical lymphocytes with prominent nucleoli and frequent mitotic figure (inset, hematoxylin and eosin, ×600) within small vessels in the deep dermis to subcutaneous tissue (hematoxylin and eosin, ×40).

**Figure 4:**
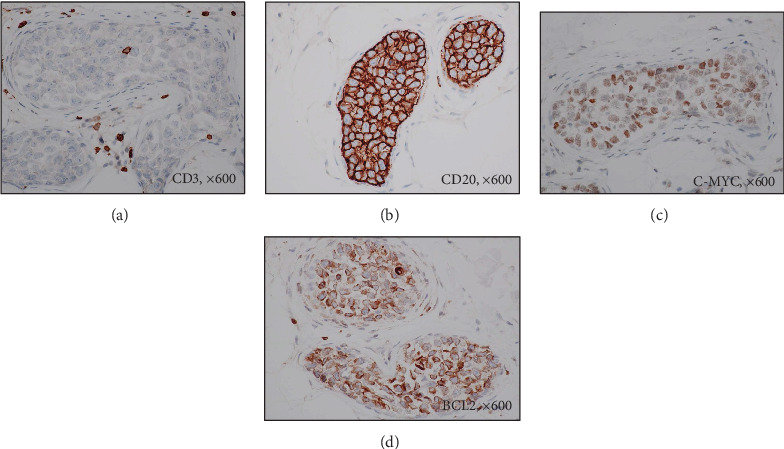
Staining of atypical lymphocytes shows negative for CD3 (a, ×600), but positive for CD20 (b, ×600), C-MYC (c, ×600), and BCL2 (d, ×600).

Table 2 shows data from skin biopsy. Of the 4 patients, excluding 1 patient who developed skin lesions after RSB, 3 patients underwent lesional skin biopsy. All specimens from lesional biopsy (1 from the abdomen, 3 from the right thigh, and 3 from the left thigh) showed a positive result. Sections of RSB obtained from the right thigh had the highest frequency of positivity (85.7%), followed by the abdomen (75%) and left thigh (71.4%). Histopathology revealed the presence of large atypical lymphocytes with prominent nucleoli and frequent mitotic figures within small vessels in the deep dermis to subcutaneous tissue (Figure 3). Confirming the diagnosis of B-cell origin, CD20 was positive while CD3 was negative in all patients (Figures 4(a) and 4(b)). The frequencies of positive results of other markers were as follows: CD10 (0%), BCL6 (66.7%), MUM-1 (73.3%), CD5 (13.3%), CD30 (6.7%), BCL2 (86.7%), C-MYC (40.0%), and P53 (73.3%) (Table 3). Classifying by Hans algorithm [13], available immunohistochemical findings in 15 patients demonstrated that 11 (73.3%) and 2 (13.3%) were categorized as nongerminal center (non-GCB) variant and germinal center B-cell- (GCB-) like variant, respectively, while 2 patients were unclassifiable. C-MYC/BCL2 double expressor was noted in 6 (40.0%) patients (Figures 4(c) and 4(d)).

### 3.4. Therapy and Therapeutic Outcomes

**Figure 5:**
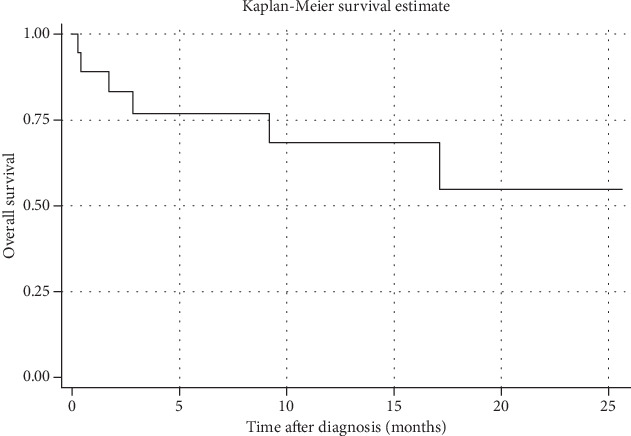
Survival curve for IVLBCL patients.

The median follow-up time was 10 months (0.5-26 months). Almost all (15 out of 17 patients) received chemotherapy as in Table 1. Two patients (patient numbers 14 and 16) preferred palliative care to aggressive management. R-CHOP (rituximab, cyclophosphamide, doxorubicin, vincristine, and prednisolone) or CHOP (cyclophosphamide, doxorubicin, vincristine, and prednisolone) regimen was given in 6 and 8 patients, respectively. One patient was treated with hyper-CVAD (cyclophosphamide, vincristine, doxorubicin, and dexamethasone) followed by ESHAP (etoposide, methylprednisolone, high-dose cytarabine, and cisplatin) regimen. Of 6 patients with R-CHOP, 5 (83.3%) achieved complete remission and are now alive without disease while 1 patient has ongoing treatment (patient number 10). Two patients (patient number 7 and 8) experienced relapse, one (patient number 7) then had progressive disease and was transferred to outside hospital, while the other (patient number 8) died within 18 months. Of all, 6 (31.6%) patients died from disease progression. From time-to-event analysis, the incidence rate to complete remission was 10.8 persons per 100 persons per month and the median time to complete remission was 7.5 months (95%CI = 5.0 − 8.1 months). One-year and two-year overall survival rates were 67.2% and 53.8%, respectively (Figure 5).

### 3.5. Characteristics in Predicting Disease Prognosis

In terms of IPI score, there was no significant difference between patients in the double expressor group and nondouble expressor group with mean values of 4.5 ± 0.22 and 4.1 ± 0.20, respectively (*p* = 0.88). Clinical characteristics, laboratory features, IPI score, or treatment regimens did not show significant association with IVLBCL disease prognosis for both complete remission and death (*p* > 0.05). Patients in the non-GCB group demonstrated a lower incidence rate to complete remission and a higher incidence rate to death than GCB-like group (8 vs. 14.4 persons per 100 persons per month and 4.5 vs. 0 person per 100 persons per month, respectively), although no statistically significant differences were found (*p* = 0.429 and *p* = 0.356, respectively). Also, patients with C-MYC/BCL2 coexpression had a lower incidence rate to complete remission (3.7 persons per 100 persons per month) when compared with nondouble expressors (11.8 persons per 100 persons per month) but this difference did not reach a statistical significant level (*p* = 0.212). C-MYC/BCL2 double expressors, however, showed a significantly higher incidence rate to mortality in comparison with the other groups, (14 vs. 1.8 persons per 100 persons per month, respectively, *p* = 0.042). The same pattern was observed in C-MYC-positive patients (Table 4). It is important to note that all patients with C-MYC expression also showed positive BCL2 in our study. A lower incidence rate in attaining complete remission and a higher incidence rate to mortality were revealed in both the CD5-positive group and CD30-positive group when compared to those with negative results; however, no statistical differences were seen. Patients with positive P53 had a slightly higher incidence rate to complete remission and mortality than those with a negative test though without significant differences. We also performed multivariate analysis for mortality including C-MYC/BCL2 double expressor, CD30, and treatment regimen (categorized as R-CHOP, CHOP, or others). However, no statistical significance was found (*p* = 0.312, *p* = 0.730, and *p* = 1.000, respectively).

## 4. Discussion

According to 2017 World Health Organization classification of tumours of hematopoietic and lymphoid tissue [3], IVLBCL is classified as a rare type of non-Hodgkin lymphoma, accounting for 0.1 to 0.91% of all non-Hodgkin's lymphoma (NHL) [18–20]. The age-adjusted incidence rate of IVLBCL was 0.095 case per 1,000,000 American persons [21]. Histology shows a proliferation of lymphoma cells within the lumen of small blood vessels, especially the capillaries [2]. Owing to extremely variable and nonspecific clinical presentations, making the diagnosis is challenging. Furthermore, due to the rarity of the condition, our understanding of the clinical course, management, and prognostic factors of IVLBCL is limited. For this reason, we focused on cases with IVLBCL diagnosed by skin biopsy, and to the best of our knowledge, our study is among the few highlighting that IHC could be utilized as a prognostic factor in IVLBCL patients [4, 22].

In agreement with previous reports [6, 18, 23, 24], we found comparable age at diagnosis (67 years old) but slightly older than other Asians [4, 11, 20]. A slight male predominance which is consistent with studies from Thailand [11, 20], Taiwan [4], and China [22] was observed in our study, although studies by Murase et al. [24] and Rajyaguru et al. [21] showed no gender preference. Fever was found in the majority of patients while neurologic abnormalities were also relatively common in this study. A case series conducted in 38 patients [5] reported fever and neurological symptoms in 17 (45%) and 13 (34%) of IVLBCL patients. Neurological symptoms varied from sensory and motor deficits, paresthesia, dysarthria, and aphasia, to altered conscious state [5]. The frequencies of neurological symptoms and cutaneous lesions in our study were consistent with studies from Italy and Thailand [5, 20] but higher than a study from Japan [24]. Unfortunately, it is difficult to conclude if our cohort fits the Asian variant since we lacked information to fulfill the criteria particularly the presence of hemophagocytic syndrome [25]. Our results confirmed the variable morphology of cutaneous manifestations as seen in other studies [2]. Regarding bone marrow study, although a recent series of IVLBCL patients diagnosed by random skin biopsy showed no BM involvement [11], we found approximately 35% with BM infiltration which is consistent with previous studies ranging from 26.7% to 100% [4, 6, 20, 22, 24, 26, 27]. Additionally, we found that increased sera LDH and ferritin were exceedingly common in IVLBCL patients, consistent with earlier publications reporting 75%-100% of elevated LDH [5, 11, 20, 22, 23, 26] and 80-100% of elevated ferritin [4, 26] in IVLBCL patients.

With regard to skin biopsy, our results showed that all patients with suspected IVLBCL, irrespective of the presence or absence of skin lesions, had positive random skin biopsy. This emphasizes that skin biopsy, particularly random skin biopsy, which is a minimally invasive method, appears to be a useful diagnostic tool even in the absence of BM invasion and sometimes can preclude unnecessary biopsy of other vital organs. Therefore, we agree with previous reports that incisional skin biopsy deep enough to include subcutaneous fat from at least 3 different fat-bearing locations is recommended [9–14]. Sections from the lower extremities and trunk are important and may yield a better chance to detect lymphoma cells since our results suggest that cutaneous lesions in IVLBCL commonly affected these areas. In cases with uncertainty in diagnosis such as those with prolonged and unexplained fever, no nodal involvement, no cutaneous lesion, increased serum LDH, and increased serum ferritin, we highly suggest performing random skin biopsy to confirm the diagnosis of IVLBCL.

Consistent with previous literature [24], patients receiving anthracycline-based chemotherapy in combination with rituximab (monoclonal anti-CD20 antibody) were found to have favorable outcomes. Ferreri et al. [28] proved that rituximab in conjunction with chemotherapy significantly improved complete remission rate (90% vs. 50%, *p* = 0.04), event-free survival (3-year: 89% vs. 35%, *p* = 0.03), and also overall survival rate (3-year: 89% vs. 38%, *p* = 0.01) compared to chemotherapy alone. However, this should be interpreted with caution since some patients suffered from poor performance status and were excluded from aggressive management.

Our overall survival rates of 67.2% and 53.8% for 1-year and 2-year were similar to that of American population-based study (1-year overall survival rate = 66.4%) [21], but relatively higher than the large western series by Ferreri et al. [5] and the recent study by Wang et al. [22], representing 30 ± 7% and 32.7% of 2-year overall survival. Other studies also noted unfavorable prognosis of 27%-67% for 3-year overall survival rate [18, 21, 23, 24]. This difference may be partially justified by different ethnicities and the fact that the previous studies included patients with either *in vivo* or postmortem diagnosis while only living cases were enrolled in the present study.

In determining the prognosis of IVLBCL, we observed no significant association between demographic data, clinical features, laboratory investigation, or therapeutic regimen with the incidence rate of complete remission or mortality. As IVLBCL is considered a type of DLBCL, and immunohistochemistry, namely CD5^+^, CD30^+^, P53^+^, C-MYC^+^, and C-MYC/BCL2 double expression have been utilized as poor prognostic prediction in DLBCL, NOS, [15] concurrent expression of C-MYC/BCL2 defined by ≥40% C-MYC+ and ≥50%-70% BCL2^+^ cells [3] has been documented as a poor prognostic factor [15], and some authors proposed that it is a more reliable method for predicting risk of DLBCL than cell-of-origin classification [29–32]. In our study, C-MYC/BCL2 double expressors showed significantly worse prognosis, specifically mortality, compared to nondouble expressors. To our knowledge, no previous study has described this association in IVLBCL patients before. As for the expression of C-MYC alone, the finding should be interpreted with caution despite statistical significance. It is crucial to note that all C-MYC-positive patients also showed positive results for BCL2. Therefore, we postulate that the expression of C-MYC in conjunction with BCL2 could be utilized as an unfavorable prognostic indicator in IVLBCL patients in order to allow physicians to give prompt and aggressive management including the initiation of rituximab to improve survival.

In terms of other prognostic indicators, a higher incidence rate to death and a lower incidence rate to remission have been found in non-GCB variant-, CD5-, and CD30-positive groups but without statistical difference. Apparently, P53 failed to predict prognosis in our study since it showed a slightly higher incidence rate in complete remission and in mortality than those with negative test with no significant statistical difference. Thus, we conclude that non-GCB variant, CD5, CD30, or P53 is not a significant prognostic predictor in IVLBCL.

The main limitation of this study is its retrospective nature and a small number of patients. Extracutaneous involvement was not histologically confirmed in every patient since all cases were diagnosed *in vivo*. Moreover, we could not classify our patients into “Classic variant” or “Asian variant” because some data to evaluate the criteria especially the presence of hemophagocytic syndrome were missing. In addition, the follow-up time is relatively short. A greater number of subjects and longer follow-up period would give more apparent effects and statistically significant differences.

## 5. Conclusion

To summarize, we demonstrate that clinical manifestations of IVLBCL are markedly variable. Skin biopsy, particularly random skin biopsy, is an effective diagnostic method and is highly recommended in patients suffering from unexplained fever or neurological symptoms especially in combination with increased LDH and serum ferritin. Lastly, concurrent expression of C-MYC and BCL2 may be a useful prognostic indicator and should be performed to help predict the prognosis in IVLBCL patients.

## Figures and Tables

**Table 1:** Summary of clinical characteristics, laboratory investigation, and therapeutic response in IVLBCL patients.

No.	Sex, age (years)	Presenting symptoms	Duration (weeks)	Skin lesions	BM involvement	Other associated symptoms	Diagnostic tissue from other organs	LDH^∗^ (U/L)	Ferritin^∗∗^ (ng/mL)	IPI score	Treatment	Result after initial treatment	Status at last follow-up	Total follow-up time (months)
1	M, 61	Fever	12	No	Yes	Dyspnea	—	592	2369	5	R-CHOP	CR	AW	8
2	M, 61	Fever	8	No	Yes	—	Lungs (bronchoalveolar lavage)	848	NA	4	CHOP	CR	AW	14
3	M, 77	Fever	5	No	No	—	—	1142	1596.2	4	CHOP	PD	DWD	0.5
4	F, 65	Fever	12	No	No	Dyspnea, motor neuropathy	—	1047	35766.4	4	R-CHOP	CR	AW	26
5	M, 57	Fever	12	No	No	—	—	811	7494	3	CHOP	CR	AW	12
6	F, 69	Fever	8	No	No	—	—	708	2123.4	4	R-CHOP	CR	AW	20
7	F, 65	Fever	12	Yes	No	Hepatosplenomegaly, alteration of consciousness	—	1154	NA	4	CHOP⟶transferred	CR⟶R⟶PD	AWD	26
8	F, 54	Fever	NA	No	Yes	Hepatosplenomegaly, cauda equina syndrome	—	NA	NA	NA	ESHAP, hyper-CVAD	PD	DWD	18
9	M, 59	Fever	4	No	No	Hepatomegaly	—	NA	NA	NA	CHOP	PD	DWD	0.5
10	F, 80	Fever	4	No	Yes	—	—	528	4629.4	5	R-CHOP	PT	AWD	4.5
11	M, 77	Fever	1	Yes	No	Splenomegaly	Maxillary and ethmoid sinus	1481	1891.2	5	CHOP	PD	DWD	3
12	F, 59	Alteration of consciousness	4	Yes	No	Dyspnea	—	317	1888	4	CHOP	PR	DWD	10
13	F, 56	Dementia	12	No	No	—	Brain	346	342.8	4	R-CHOP	CR	AW	21
14	M, 77	Progressive paralysis	8	No	No	Dyspnea	—	565	NA	4	Transferred	Transferred	AWD	4
15	M, 83	Alteration of consciousness	8	Yes	Yes	—	—	6763	2582.1	5	CHOP	PD	DWD	2
16	M, 64	Abdominal pain and jaundice	2	No	Yes	Hepatosplenomegaly	—	1362	NA	5	Transferred	Transferred	AWD	0.5
17	M, 81	Anorexia	8	No	No	—	—	1246	2505	4	R-CHOP	CR	AW	18

Abbreviations used: BM: bone marrow; IPI: international prognostic index; LDH: lactate dehydrogenase; M: male; F: female; NA: data not available; R-CHOP = rituximab, cyclophosphamide, doxorubicin, vincristine, and prednisolone; CHOP: cyclophosphamide, doxorubicin, vincristine, and prednisolone; ESHAP: etoposide, methylprednisolone, high-dose cytarabine, and cisplatin; Hyper-CVAD: cyclophosphamide, vincristine, doxorubicin, and dexamethasone; CR: complete remission; PD: progressive disease; PT: in the period of treatment; R: relapse; PR: partial remission; AW: alive and well; DWD: dead with disease; AWD: alive with disease. ^∗^Normal serum LDH = 0 − 220 U/L. ^∗∗^Normal serum ferritin = 4.6 − 204 ng/mL.

**Table 2:** Frequencies of positive skin biopsy according to location.

Location	Lesional skin biopsy Positive, *N* (%)	Random skin biopsy^∗^ Positive, *N* (%)
Abdomen	1/1 (100.0)	12/16 (75.0)
Right thigh	3/3 (100.0)	12/14 (85.7)
Left thigh	3/3 (100.0)	10/14 (71.4)

^∗^One patient developed skin lesion after random skin biopsy.

**Table 3:** Immunochemical findings and cell-of-origin classification.

Patient number	CD3	CD20	CD10	BCL6	MUM-1	Cell-of-origin classification	CD5	CD30	BCL2	C-MYC	P53
1	-	+	-	+	-	GCB-like	-	-	+	-	+
2	-	+	NA	NA	NA	NA	NA	NA	NA	NA	NA
3	-	+	-	-	+	Non-GCB-like	-	-	+	+	+
4	-	+	-	+	+	Non-GCB-like	-	-	+	-	+
5	-	+	-	+	+	Non-GCB-like	-	-	+	-	+
6	-	+	NA	NA	NA	NA	NA	NA	NA	NA	NA
7	-	+	-	+	-	GCB-like	-	-	+	-	-
8	-	+	-	-	-	Unclassifiable	-	-	-	-	-
9	-	+	-	-	-	Unclassifiable	-	-	-	-	-
10	-	+	-	+	+	Non-GCB-like	-	-	+	+	+
11	-	+	-	+	+	Non-GCB-like	+	-	+	+	+
12	-	+	-	+	+	Non-GCB-like	-	-	+	+	+
13	-	+	-	+	+	Non-GCB-like	-	-	+	+	+
14	-	+	-	-	+	Non-GCB-like	-	-	+	-	-
15	-	+	-	+	+	Non-GCB-like	-	-	+	+	+
16	-	+	-	-	+	Non-GCB-like	-	-	+	-	+
17	-	+	-	+	+	Non-GCB-like	+	+	+	-	+
Positive (%)	0	100.0	0	66.7	73.3	-	13.3	6.7	86.7	40.0	73.3

GCB: germinal center B-cell; Non-GCB = nongerminal center B-cell; NA: no data available.

**Table 4:** Association between histochemical expression and incidence rate to complete remission and mortality.

Characteristics	"Complete remission rate" (persons per 100 persons per month)		*p* value	"Mortality rate" (persons per 100 persons per month)		*p* value
	Positive	Negative		Positive	Negative	
Non-GCB-like variant	8.0	14.4	0.429	4.5	0.0	0.356
C-MYC/BCL2 double expressor	3.7	11.8	0.212	14.0	1.8	0.042^∗^
CD5^+^	8.9	9.6	0.892	4.8	4.2	0.985
CD30^+^	0.0	9.2	1.000	57.5	3.7	0.086
C-MYC^+^^∗∗^	3.7	11.8	0.213	14.0	1.8	0.042^∗^
P53^+^	9.4	8.3	0.549	4.4	4.3	0.901

^∗^Statistical significance. ^∗∗^All patients with positive C-MYC showed positive result for BCL2.

## Data Availability

The data used to support the findings of this study are included within the article.
